# Clinical Usefulness of Computational Modeling-Guided Persistent Atrial Fibrillation Ablation: Updated Outcome of Multicenter Randomized Study

**DOI:** 10.3389/fphys.2019.01512

**Published:** 2019-12-17

**Authors:** In-Soo Kim, Byounghyun Lim, Jaemin Shim, Minki Hwang, Hee Tae Yu, Tae-Hoon Kim, Jae-Sun Uhm, Sung-Hwan Kim, Boyoung Joung, Young Keun On, Seil Oh, Yong-Seog Oh, Gi-Byung Nam, Moon-Hyoung Lee, Eun Bo Shim, Young-Hoon Kim, Hui-Nam Pak, for the CUVIA-AF1 Investigators

**Affiliations:** ^1^Yonsei University Health System, Seoul, South Korea; ^2^Cardiovascular Center, Korea University, Seoul, South Korea; ^3^Division of Cardiology, Catholic University of Korea, Seoul, South Korea; ^4^Samsung Medical Center, Sungkyunkwan University, Seoul, South Korea; ^5^Division of Cardiology, Seoul National University, Seoul, South Korea; ^6^Asan Medical Center, University of Ulsan, Ulsan, South Korea; ^7^Department of Mechanical and Biomedical Engineering, Kangwon National University, Chuncheon, South Korea

**Keywords:** atrial fibrillation, catheter ablation, virtual ablation, computational modeling, recurrence

## Abstract

**Objective:**

Catheter ablation of persistent atrial fibrillation (AF) is still challenging, no optimal extra-pulmonary vein lesion set is known. We previously reported the clinical feasibility of computational modeling-guided AF catheter ablation.

**Methods:**

We randomly assigned 118 patients with persistent AF (77.8% men, age 60.8 ± 9.9 years) to the computational modeling-guided ablation group (53 patients) and the empirical ablation group (55 patients) based on the operators’ experience. For virtual ablation, four virtual linear and one electrogram-guided lesion sets were tested on patient heart computed tomogram-based models, and the lesion set with the fastest termination time was reported to the operator in the modeling-guided ablation group. The primary outcome was freedom from atrial tachyarrhythmias lasting longer than 30 s after a single procedure.

**Results:**

During 31.5 ± 9.4 months, virtual ablation procedures were available in 95.2% of the patients (108/118). Clinical recurrence rate was significantly lower after a modeling-guided ablation than after an empirical ablation (20.8 vs. 40.0%, log-rank *p* = 0.042). Modeling-guided ablation was independently associated with a better long-term rhythm outcome of persistent AF ablation (HR = 0.29 [0.12–0.69], *p* = 0.005). The rhythm outcome of the modeling-guided ablation showed better trends in males, non-obese patients with a less remodeled atrium (left atrial dimension < 50 mm), ejection fraction ≥ 50%, and those without hypertension or diabetes (*p* < 0.01). There were no significant differences between the groups for the total procedure time (*p* = 0.403), ablation time (*p* = 0.510), and major complication rate (*p* = 0.900).

**Conclusion:**

Among patients with persistent AF, the computational modeling-guided ablation was superior to the empirical catheter ablation regarding the rhythm outcome.

**Clinical Trial Registration:**

This study was registered with the ClinicalTrials.gov, number NCT02171364.

## Introduction

Catheter ablation is an effective and established rhythm control strategy in patients with anti-arrhythmic drug-resistant atrial fibrillation (AF) (Calkins et al., 2012). Nevertheless, catheter ablation of persistent AF (PeAF) or longstanding PeAF is a challenging procedure, and the post-procedural recurrence rate is still substantially high (Verma et al., 2015). This is because extra-pulmonary vein (PV) triggers are more common in patients with PeAF (Pak et al., 2006), but they cannot be treated with circumferential PV isolation (CPVI) alone. Thus, empirical extra-PV ablation for substrate modification strategies has been applied for patients with PeAF (Willems et al., 2006) and has been shown to be beneficial (Knecht et al., 2008). Linear ablation may improve the rhythm outcome by a reduction in the critical mass and exit block of extra-PV triggers, despite the difficulty to obtain long-lasting bidirectional block of linear lesions (Kim et al., 2016). Whereas, a randomized clinical trial failed to prove the beneficial effect of empirical extra-PV ablation compared to the CPVI in patients with PeAF (Verma et al., 2015). Therefore, personalized, focused, and effective ablation strategies are required, rather than a routine empirical extra-PV left atrial (LA) ablation with extensive cardiac tissue damage.

Recently, computational modeling has been making remarkable progress, and its clinical usefulness is gradually increasing. Computational AF modeling reflecting a sophisticated histology such as fibrosis and a personalized fiber orientation using magnetic resonance imaging (MRI)-late gadolinium enhancement has been developed and is now applicable to clinical data (Jacquemet, 2015; Trayanova et al., 2018). Because the wave-dynamics mechanism of AF is highly affected by the atrial anatomy and surface curvature (Song et al., 2018), we hypothesized that a customized extra-PV ablation according to the anatomy of the atrium would reduce the recurrence rate after PeAF or longstanding PeAF ablation. The purpose of this study was to evaluate the effect of a computational modeling-guided AF ablation to improve the rhythm outcome of PeAF ablation as compared to an empirical treatment conducted by experts. Five different linear and electrogram-guided virtual ablation methods were tested with an AF modeling reflecting the patients left atrial (LA) anatomy obtained from heart computed tomography (CT) images before the procedure. The pre-determined lesion set with the best virtual AF termination was provided to operators of the computational modeling-guided ablation group; however, the operators of the empirical ablation group were blinded to the simulation outcome.

## Materials and Methods

### Study Design and Population

This study was a randomized, open-label, multicenter trial involving patients with non-valvular AF undergoing catheter ablation (ClinicalTrials.gov; NCT 02171364). The study protocol adhered to the Declaration of Helsinki and was approved by the institutional review board of each participating center. Written informed consent was obtained from all patients. The study design and results of the CUVIA-AF 1 trial have been reported (Shim et al., 2017). The study cohort included 118 patients (77⋅8% men, age 60⋅8 ± 9⋅9 years) who underwent catheter ablation of symptomatic and drug-refractory non-valvular PeAF at six tertiary hospitals in Korea. Key exclusion criteria were as follows: (1) patients younger than 20 or older than 80 years; (2) valvular AF; (3) significant structural heart disease other than left ventricular hypertrophy; (4) an LA diameter of 60 mm or more, and (5) a history of previous AF ablation or cardiac surgery. The anatomy of the LA and PVs was visually defined by three-dimensional (3D) CT scans (64 Channel, Light Speed Volume CT, Philips, Brilliance 63, Amsterdam, Netherlands). All anti-arrhythmic drugs (AADs) were discontinued for a period of at least five half-lives.

**FIGURE 1:**
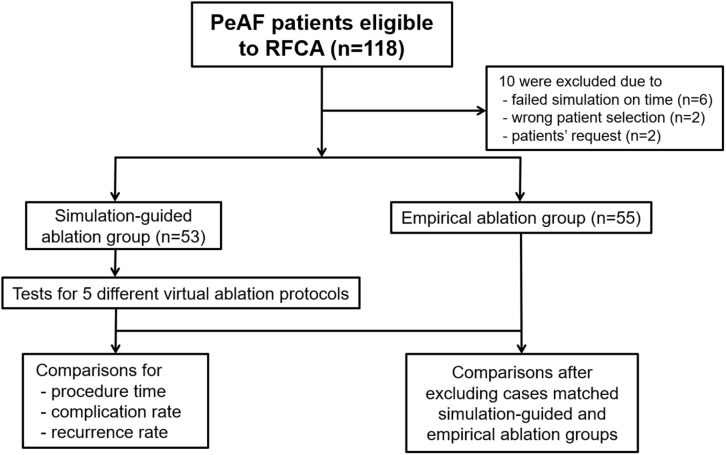
Study flow diagram. The enrolled patients were randomly assigned to either the computational modeling-guided ablation group or the empirical ablation group. PeAF, persistent atrial fibrillation; RFCA, radiofrequency catheter ablation.

The enrolled patients were randomly assigned to either the computational modeling-guided ablation group or the empirical ablation group (Figure 1). For all patients in both groups, digital images and communication in medicine (DICOM) files of the cardiac CT images were sent to the core lab, and AF modeling and virtual ablation tests were conducted before the clinical AF ablation. The ablation lesion sets were categorized by applying the following five strategies: (1) CPVI alone, (2) CPVI and an additional posterior box lesion (POBI) (Hwang et al., 2014; Kim et al., 2016), (3) additional POBI and an anterior linear (AL) ablation (Shim et al., 2017), (4) additional roof line (RL) and a left lateral isthmus (LLI) line (Haissaguerre et al., 2005), and (5) an additional complex fractionated atrial electrogram (CFAE)-guided ablation (Nademanee et al., 2004; Hwang et al., 2014). Among the five different ablation lesions, the best virtual ablation lesion set was determined by the earliest termination of the virtual AF with the simulation modeling study. For patients in the computational modeling-guided ablation group, the operator was informed about the best virtual ablation strategy and applied the clinical AF ablation procedure. In the empirical ablation group, the ablation lesion set was selected by the operator based on his or her experience. The primary end point was AF recurrence after a single procedure, and the secondary end point was a recurrence pattern, the response to AADs, and the cardioversion rate. All ablation procedures were performed by physicians with at least 10 years of experience.

### Computational Modeling of AF

After receiving consent from the patients, DICOM files of the heart CT images were sent to the core laboratory the night before or early in the morning of the AF ablation procedure. The patient randomization, computational AF modeling, and virtual AF ablation were performed at the core laboratory during working hours (9 a.m.–6 p.m.). Among 118 patients, four were excluded for incorrect selection and six were excluded due to a failed virtual AF ablation due to DICOM file errors or communication errors. Finally, 108 patients were enrolled and randomly assigned into two groups: 53 in the computational modeling-guided ablation group and 55 in the empirical ablation group (Figure 1). The patient characteristics are shown in Table 1.

**TABLE 1:** Baseline clinical characteristics.

	**Overall (*N* = 108)**	**Simulation-guided ablation (*N* = 53)**	**Empirical ablation (*N* = 55)**	***p*-Value**
Age (years)	60.8 ± 9.6	59.7 ± 10.1	61.9 ± 9.6	0.240
Male, *n* (%)	(76.9%)	(75.5%)	(78.2%)	0.821
Longstanding persistent AF (%)	(77.8%)	(83.0%)	(72.7%)	0.249
AF duration	44.1 ± 55.6	39.4 ± 58.1	48.3 ± 53.5	0.441
Follow-up duration, months	31.5 ± 9.4	31.7 ± 9.3	31.3 ± 9.5	0.830
BMI (kg/m^2^)	25.3 ± 3.1	25.7 ± 3.5	24.8 ± 2.6	0.129
CHA_2_DS_2_–VASc score	2.0 ± 1.9	1.9 ± 1.7	2.1 ± 1.9	0.475
Congestive heart failure (%)	(12.0%)	(9.4%)	(14.5%)	0.557
Hypertension (%)	(54.6%)	(52.8%)	(56.4%)	0.847
Age ≥ 75 years (%)	(9.3%)	(3.8%)	(14.5%)	0.094
Age 65–74 years (%)	(25.0%)	(28.3%)	(21.8%)	0.508
Diabetes (%)	(18.5%)	(17.0%)	(20.0%)	0.806
Previous stroke (%)	(28.7%)	(28.3%)	(29.1%)	>0.999
Previous TIA (%)	(1.9%)	(3.8%)	(0.0%)	0.238
Vascular disease (%)	(13.0%)	(9.4%)	(16.4%)	0.392
Echocardiographic parameters (Pre-RFCA)				
LA diameter (mm)	45.1 ± 4.4	46.1 ± 7.6	44.0 ± 4.4	0.086
LA volume index (mL/m^2^)	44.4 ± 14.8	45.0 ± 15.7	43.8 ± 14.0	0.718
LV EF (%)	59.3 ± 9.7	57.8 ± 7.8	60.7 ± 9.7	0.092
E/Em	10.2 ± 4.7	9.6 ± 3.0	10.7 ± 4.7	0.139

*AAD, antiarrhythmic drug; ACEi, angiotensin-converting-enzyme inhibitor; ARB, angiotensin receptor blocker; BMI, body mass index; BSA, body surface area; E, early diastolic transmitral flow velocity; Em, early diastolic mitral annular velocity; ECV, electrical cardioversion; LA, left atrium; L-PeAF, long-standing persistent atrial fibrillation; LV EF, left ventricular ejection fraction; RFCA, radiofrequency catheter ablation; TIA, transient ischemic attack.*

The LA geometries of the patients included were reconstructed from the 3D CT DICOM files defining the surface of the LA. A triangular mesh was generated with a prism-type element applying a constant thickness of 1.89 mm on the surface of the 3D LA geometry (Beinart et al., 2011; Hwang et al., 2014). The final number of grid elements was set between 400,000 and 500,000. The LA appendage structure and myocardial sleeves of the LA (Pashakhanloo et al., 2016) were also included in the mesh. For the computational modeling of the cardiac wave propagation in the atrial wall, the following reaction-diffusion equation was used (Zozor et al., 2003):

(1) $$\frac{\partial V_{m}}{\partial t}=\frac{1}{\beta \,C_{m}}\,\left\{\nabla \cdot D\,\nabla V_{m}-\beta \,\left(I_{\mathrm{ion}}+I_{s}\right)\right\},$$

where *Vm* (volt) is the membrane potential; β (meter^–1^) is the membrane surface-to-volume ratio; *Cm* (farad/meter^2^) is the membrane capacitance per unit area; *D* (siemens/meter) is the conductivity tensor; and *I*_*ion*_ and *I*_*s*_ (ampere/meter^2^) are the ion current and stimulation current, respectively. To simulate the reaction-diffusion system, we constructed the models using a generalized finite difference scheme which can effectively lower dimensionality and can reduce computing time with parallel computational modeling with graphics processing unit system (Zozor et al., 2003).

For the calculation of ionic currents, a mathematical model of the human atrial action potential was used (Courtemanche et al., 1998). Electrical stimulation was applied at the location of Bachmann’s bundle, and reentry was initiated by rapid pacing: a total of 24 paces (eight paces per each pacing cycle length) with pacing cycle lengths of 200, 190, and 180 ms. The ionic currents in each cell were determined using the human atrial myocyte model applied by modified model from that of Courtemanche et al. (1998) To replicate the electrical remodeling associated with AF in the cell model, the conductances of I_*to*_, I_*CaL*_, I_*Kur*_, and I_*K*__1_ were changed by −80, −40, −50, and +50%, respectively, as described previously by us (Shim et al., 2017) and others (Dossel et al., 2012; Wilhelms et al., 2012). We chose a conduction velocity (CV) of 0.4 m/s based on human patient data (Yonsei AF ablation cohort data; *n* = 1,980; mean CV = 0.43 ± 0.24 m/s) (Park et al., 2014) and previous modeling studies (Hwang et al., 2014).

### Virtual AF Ablation

**FIGURE 2:**
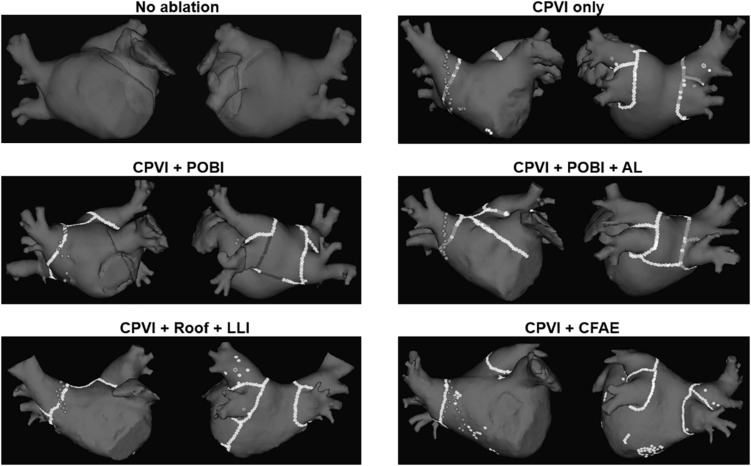
Five different protocols of virtual ablation. AL, left atrial anterior linear line; CFAE, complex fragmented atrial electrogram; CPVI, circumferential pulmonary vein isolation; LLI, left atrial left lateral isthmus line; POBI, posterior box isolation; Roof, left atrial roof line.

Virtual ablation was performed for all 108 patients in both the computational modeling-guided and empirical ablation groups. We developed a graphical user interface software, which had already been introduced (Shim et al., 2017), with which the user can perform virtual ablation by mouse-clicking on the atrial geometry (CUVIA, Model: SH01, ver. 1.0; Laonmed, Inc., Seoul, South Korea). The ablation patterns of each of the five protocols are shown in Figure 2. At the ablated lesion points, the membrane potential was permanently set to the resting value (−80.6 mV) to generate conduction block. For the CFAE-guided ablation, the areas of CFAEs with a cycle length (CFAE CL) of less than 120 ms were ablated starting from the lowest CFAE CL area as long as the total ablated area was less than 5% of the total atrial area. Mitral annular area was set as non-conductive area. Virtual ablation was applied 4 s after the end of pacing. Computational modeling was applied for 25 s, and the duration lasted until the fibrillation termination was recorded for each ablation protocol. Virtual AF termination was determined as the time to the total extinction of the wavelet (90% repolarization) in the entire LA. A graphical user interface was developed using the C++ order to perform each virtual ablation on the virtual atrium of each patient.

### Clinical AF Ablation

Electrophysiological mapping and radiofrequency catheter ablation (RFCA) have been described previously (Shim et al., 2017). Briefly, we used an open irrigated-tip catheter (Thermocool [Johnson & Johnson, Inc., Diamond Bar, CA, United States] or Coolflex [St. Jude Medical]; 30–35W, 47°C) to deliver radiofrequency energy for the ablation under 3D electroanatomical mapping (NavX, St. Jude Medical; CARTO3, Johnson & Johnson) merged with the 3D spiral CT. All patients underwent a CPVI in both groups. After CPVI, bidirectional block was confirmed in all patients. Extra-PV ablation was performed based on the virtual ablation outcome in the computational modeling-guided ablation group and was based on the operator’s discretion in the empirical ablation group. The procedure was completed when there was no immediate recurrence of AF after cardioversion with an isoproterenol infusion (up to 5 μg/min). All RFCA procedures were performed by an operator with over 10 years of experience.

### Post-ablation Management and Follow-Up

After the procedure, we maintained AADs for 3 months in 58.3% of the included patients, as the majority had longstanding persistent AF (78% of included patients). Then, we tried to stop AAD if there was no recurred AF in the post-procedure 3^*rd*^ month Holter. Patients visited the outpatient clinic at one, three, six, and 12 months after the RFCA or whenever symptoms occurred. All patients underwent electrocardiography at each visit, and 24-h Holter recording was performed at 3, 6, and 12 months, and every 6 months thereafter, according to the 2012 Heart Rhythm Society/European Heart Rhythm Association/European Cardiac Arrhythmia Society Expert Consensus Statement (Calkins et al., 2012). When patients reported palpitations, Holter or event monitor recordings were obtained to check for arrhythmia recurrence. We defined recurrence as any episode of AF or atrial tachycardia (AT) lasting for at least 30 s. Any electrocardiography documentation of an AF recurrence after a 3 months blanking period was diagnosed as a clinical recurrence.

### Statistical Analysis

Continuous variables were compared with a Student’s *t*-test, and categorical variables were compared with either the chi-square test or Fisher’s exact test, as appropriate. The primary endpoint was the freedom from any atrial arrhythmias during the follow-up after a 3- month blanking period. The time to recurrence and arrhythmia-free survival were assessed with a Kaplan–Meier analysis, and differences were calculated with the log-rank test. To assess the factors associated with a post-RFCA clinical recurrence of AF, we used a Cox proportional-hazard model regression analysis. *p*-Values of < 0.05 were considered statistically significant. All statistical analyses were performed using SPSS version 25.0 software (SPSS, Inc., Chicago, IL, United States).

## Results

### Baseline Characteristics

We enrolled 118 patients with persistent AF for catheter ablation in the CUVIA AF trial. Among them, 10 were excluded due to a failed virtual ablation before starting the clinical procedure (*n* = 6), wrong patient selection (*n* = 2), or upon patient request (*n* = 2); finally, 108 patients (95.2%) were randomized (Figure 1). All included patients initiated the clinical AF ablation procedure after finishing the virtual AF ablation. Eventually, 53 patients were properly and randomly allocated to the computational modeling-guided ablation group, and 55 were allocated to the empirical ablation group (Figure 1). The patient characteristics are summarized in Table 1. The mean age was 61 years, 77% were male, and the overall follow-up duration was 31.5 months. The proportion of longstanding PeAF (sustaining more than 1 year) was 78% (83.0% in the modeling-guided ablation group vs. 72.7% in the empirical ablation group, *p* = 0.249). The mean CHA_2_DS_2_-VASc score was 2.0 ± 1.9 and mean LA dimension 45.1 ± 4.4 mm. There were no statistically significant differences in the patient characteristics between the groups.

### Virtual Ablation Outcome

Per the study protocol, a virtual ablation was performed in all included patients before starting the clinical AF ablation; however, the results were blinded for the empirical ablation group. The pre-ablation computing time for simulating the five virtual ablation strategies described previously was 166 ± 11 min. One hour was required for the heart CT segmentation with a semi-automatic method and settings of five different virtual ablation lesion sets by the manual method. Computing the virtual AF induction and five protocol-based virtual AF ablation lesions required one more hour, and an additional 30 min was required for calculating the CFAE area. The outcomes of the virtual AF ablation are summarized in Table 2. We determined the virtual AF termination by waiting 25 s after the virtual AF ablation. Among the five different virtual ablation lesion sets, the POBI and AL ablation after the CPVI [CPVI + POBI + AL] had the highest AF termination rate (81.5%, *p* < 0.001 vs. CPVI alone) and shortest time to AF termination (16.8 ± 5.7 s). Virtual ablation of the RL and LLI after the CPVI [CPVI + RL + LLI] had the second highest termination rate (73.1%, *p* < 0.001 vs. CPVI alone) within 25 s. The virtual AF termination rates were followed by a POBI after the CPVI [CPVI + POBI] (28.7%, *p* < 0.001 vs. CPVI), CPVI alone (11.1%), and CFAE ablation after the CPVI [CPVI + CFAE] (8.3%, Table 2). There was no significant difference in the virtual ablation outcome between the [CPVI + CFAE] and [CPVI alone] groups.

**TABLE 2:** Virtual ablation outcomes.

	**Overall (*N* = 108)**	**Simulation-guided ablation (*N* = 53)**	**Empirical ablation (*N* = 55)**	***p*-Value**
Conduction velocity (m/s)	0.41 ± 0.11	0.40 ± 0.07	0.41 ± 0.14	0.615
APD_90_ (ms)	213 ± 2	213 ± 2	213 ± 3	0.618
AF termination rate (%)				
CPVI	11.1 (12/108)	9.4 (5/53)	12.7 (7/55)	0.761
CPVI + POBI	28.7 (31/108) ^∗^	34.0 (18/53) ^∗^	23.6 (13/55)	0.289
CPVI + POBI + AL	81.5 (88/108) ^∗^	83.0 (44/53) ^∗^	80.0 (44/55) ^∗^	0.806
CPVI + RL + LLI	73.1 (79/108) ^∗^	75.5 (40/53) ^∗^	70.9 (39/55) ^∗^	0.667
CPVI + CFAE	8.3 (9/108)	7.5 (4/53)	9.1 (5/55)	1
Time to AF termination (ms)				
CPVI	23914 ± 3466	24017 ± 3354	23815 ± 3599	0.763
CPVI + POBI	21893 ± 5471	21463 ± 5719	22307 ± 5240	0.426
CPVI + POBI + AL	16792 ± 5672	16478 ± 5750	17094 ± 5633	0.575
CPVI + RL + LLI	17701 ± 5770	17199 ± 5949	18185 ± 5604	0.378
CPVI + CFAE	24170 ± 3041	24319 ± 2686	24018 ± 3385	0.619

*^∗^*p* < 0.001 compared with the CPVI of each group.APD_90_, action potential duration of 90% repolarization; CPVI, circumferential pulmonary vein isolation; POBI, posterior box isolation; AL, anterior line; RL, roof line; LLI, left lateral isthmus line; CFAE, complex fractionated atrial electrogram guided ablation.*

### Comparison of Procedural Characteristics

The procedural results and clinical outcomes are summarized in Table 3. The total procedure times, ablation times, and complication rates did not significantly differ between the groups. The AAD maintenance rates did not significantly differ between the two groups (45.3% in the computational modeling-guided ablation group vs. 32.7% in the empirical ablation group, *p* = 0.184). Compared to the modeling-guided ablation group, the ablation lesion sets with the CPVI alone were more common (30.9 vs. 1.9%, *p* < 0.001), while additional RL and LLI ablation after the CPVI [CPVI + RL + LLI] was less common (23.6 vs. 43.4%, *p* = 0.041) in the empirical ablation group. The bidirectional block rates for each linear ablation lesion did not significantly differ between the two groups (Table 3).

**TABLE 3:** Procedure-related characteristics and the clinical rhythm outcomes.

	**Overall (*N* = 108)**	**Simulation-guided ablation (*N* = 53)**	**Empirical ablation (*N* = 55)**	***p*-Value**
Procedure time (min)	264 ± 89	256 ± 69	272 ± 105	0.403
Ablation time (sec)	5122 ± 2575	4955 ± 2804	5273 ± 2368	0.510
Fluoroscopic time (min)	57 ± 30	59 ± 31	55 ± 30	0.523
Complication rate,% (n)	4.2% (4/108)	4.4% (2/53)	4.0% (2/55)	0.900
AAD utilization rate at discharge,% (n)	58.3% (63/108)	64.2% (34/53)	52.7% (29/55)	0.233
AAD utilization rate after 3 months,% (n)	53.7% (58/108)	64.2% (34/53)	43.6% (24/55)	0.108
AAD utilization at clinical recurrence,% (n)	38.9% (42/108)	45.3% (24/53)	32.7% (18/55)	0.184
Class Ic AAD,% (n)	22.2% (24/108)	28.3% (15/53)	16.4% (9/55)	0.138
Class III AAD,% (n)	18.5% (20/108)	18.9% (10/53)	18.2% (10/55)	0.928
Procedural lesion set,% (n)				
CPVI	16.7% (18/108)	1.9% (1/53)	30.9% (17/55)	**<0.001**
CPVI + POBI	6.5% (7/108)	11.3% (6/53)	1.8% (1/55)	0.058
CPVI + POBI + AL	38.0% (41/108)	39.6% (21/53)	36.4% (20/55)	0.843
CPVI + RL + LLI	33.3% (36/108)	43.4% (23/53)	23.6% (13/55)	**0.041**
CPVI + CFAE	5.6% (6/108)	3.8% (2/53)	7.3% (4/55)	0.679
Bidirectional block rates of linear lesions				
POBI,% (n)	56.3% (27/48)	55.6% (15/27)	57.1% (12/21)	0.914
RL,% (n)	78.6% (66/84)	72.0% (36/50)	83.3% (30/34)	0.157
AL,% (n)	85.4% (35/41)	76.2% (16/21)	95.0% (19/20)	0.093
LLI,% (n)	44.4% (16/36)	39.1% (9/23)	53.8% (7/13)	0.408
Early recurrence,% (n)	31.5% (34/108)	28.3% (15/53)	34.5% (19/55)	0.490
Clinical recurrence,% (n)	30.6% (33/108)	20.8% (11/53)	40.0% (22/55)	**0.030**
CPVI	22.2% (4/18)	0% (0/1)	23.5% (4/17)	0.468
CPVI + POBI	0% (0/7)	0% (0/6)	0% (0/1)	-
CPVI + POBI + AL	26.8% (11/41)	23.8% (5/21)	30.0% (6/20)	0.664
CPVI + RL + LLI	41.7% (15/36)	21.7% (5/23)	76.9% (10/13)	**<0.001**
CPVI + CFAE	16.7% (1/6)	0% (0/2)	25.0% (1/4)	0.541
Clinical recurrence as AT,% (n)	33.3% (11/33)	9.1% (1/11)	45.5% (10/22)	**0.038**
Clinical recurrence requiring cardioversion,% (n)	16.7% (18/108)	15.1% (8/53)	18.2% (10/55)	0.670
Final sinus rhythm,% (n)	93.5% (101/108)	98.1% (52/53)	89.1% (49/55)	0.058
Final sinus rhythm without AADs,% (n)	58.3% (63/108)	52.8% (28/53)	63.6% (35/55)	0.259

*AAD, antiarrhythmic drug; AL, anterior line; CFAE, complex fractionated atrial electrogram guided ablation; CPVI, circumferential pulmonary vein isolation; LLI, left lateral isthmus line; POBI, posterior box isolation; RL, roof line. Bold values indicate statistical significance at *p* < 0.05.*

### Primary Outcome

**FIGURE 3:**
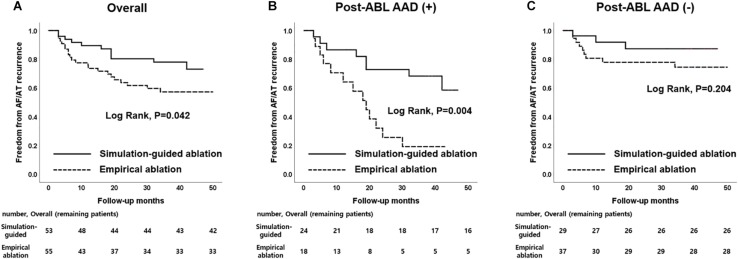
Kaplan–Meier curves according to patients with AAD usage. **(A)** Overall patients. **(B)** Patients with maintaining AAD use after catheter ablation. **(C)** Patients without maintaining AAD use after catheter ablation. AAD, antiarrhythmic drug.

**FIGURE 4:**
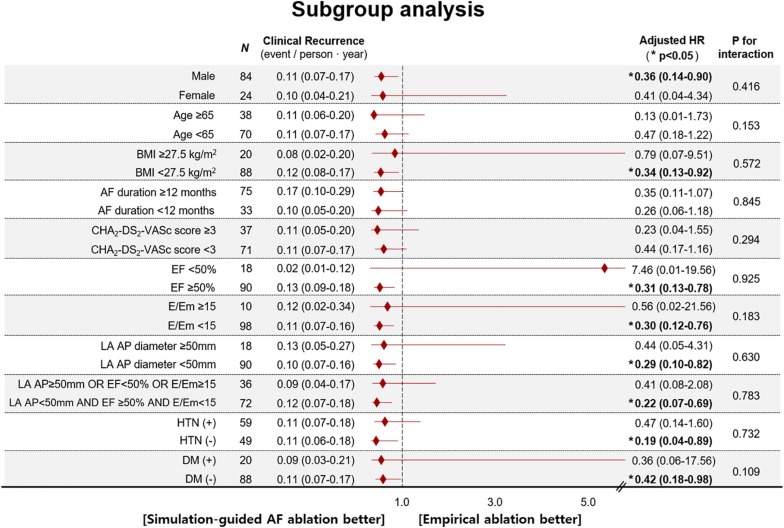
Age- and sex-adjusted HR for post-RFCA clinical recurrence of AF according to subgroups (Cox proportional-hazard model regression analysis). AF, atrial fibrillation; AP diameter, antero-posterior diameter; BMI, body mass index; DM, diabetes mellitus; EF, ejection fraction; E/Em, the ratio of early transmitral flow velocity (E) to early mitral annular velocity (Em); HR, hazard ratio; HTN, hypertension.

During the 37.1 ± 11.6 months of follow-up, the early recurrence rate within 3 months after the procedure did not significantly differ between the computational modeling-guided ablation group (28.3%) and empirical ablation group (34.5%, *p* = 0.490). However, the clinical recurrence rate after 3 months of catheter ablation was significantly lower in the modeling-guided ablation group (20.8%) than in the empirical ablation group (40.0%, *p* = 0.030, Table 3). A Kaplan–Meier analysis showed a significantly lower recurrence of AF/AT in the modeling-guided ablation group than in the empirical ablation group (log-rank *p* = 0.042, Figure 3A). This difference was significant in the patients who were taking AADs after the catheter ablation (log-rank *p* = 0.004, Figure 3B), but not in those without AADs (log-rank *p* = 0.204, Figure 3C). The presence of bidirectional block of the linear ablation lesions did not affect the clinical recurrence rate (23.1% in the modeling-guided group vs. 39.4% in the empirical ablation group, *p* = 0.138). Using a multivariable-adjusted Cox proportional hazards model regression analysis, compared with the empirical AF ablation, we found that the computational modeling-guided AF ablation reduced the clinical recurrence rate by 71% (HR 0.29 [0.12–0.69], *p* = 0.005, Table 4). In the sub-group analyses, the computational modeling-guided AF ablation showed better trends of rhythm outcome in the male individuals, non-obese individuals, patients with a left ventricular ejection fraction of ≥ 50%, E/Em of < 15, LA dimension of < 50 mm, and those without hypertension or diabetes (Figure 4).

**TABLE 4:** Factors associated with a post-RFCA clinical recurrence of AF (Cox proportional-hazard model regression analysis).

**Variables**	**Univariable adjusted**		**Multivariable adjusted**	
	**HR (95% CI)**	***p*-Value**	**HR (95% CI)**	***p*-Value**
Male	0.86 (0.35–2.07)	0.728	0.98 (0.37–2.63)	0.966
Age (years)	0.99 (0.96–1.03)	0.859	0.97 (0.93–1.01)	0.128
AF duration	1.00 (0.99–1.01)	0.397	1.00 (0.99–1.01)	0.608
CHA_2_-DS_2_-VASc	0.97 (0.79–1.19)	0.750		
Heart failure	0.41 (0.10–1.71)	0.220		
Hypertension	1.19 (0.60–2.38)	0.617		
Diabetes	0.70 (0.27–1.82)	0.467		
Previous stroke/TIA	1.25 (0.54–2.88)	0.603		
Vascular disease	0.84 (0.30–2.40)	0.747		
Baseline LA AP diameter (mm)	1.01 (0.96–1.06)	0.714	1.02 (0.97–1.07)	0.523
Baseline LV EF (%)	1.01 (0.97–1.06)	0.663		
Baseline E/Em	0.88 (0.73–1.02)	0.108		
Post-RFCA AAD use	2.09 (0.92–4.30)	0.082	2.46 (0.57–4.63)	0.102
**Simulation-guided ablation**	0.48 (0.23–0.99)	**0.048**	0.29 (0.12–0.69)	**0.005**

*AAD, antiarrhythmic drug; CI, confidence interval; E, early diastolic transmitral flow velocity; Em, early diastolic mitral annular velocity; HR, hazard ratio; LA AP, left atrial anterior–posterior; LV EF, left ventricular ejection fraction; PeAF, persistent atrial fibrillation; RFCA, radiofrequency catheter ablation; TIA, transient ischemic attack; simulation-guided ablation, virtual *in silico* model guided catheter ablation. Bold values indicate statistical significance at *p* < 0.05.*

### Secondary Outcome

Among a total of 33 patients with clinical recurrence, 22 had AF and 11 had AT at the time of the recurrence. Among the patients with clinical recurrence, the proportion of AT (9.1% [1/11] vs. 45.5% [10/22], *p* = 0.038) were lower in computational modeling-guided ablation group, but those requiring cardioversion (72.7% [8/11] vs. 45.5% [10/22], *p* = 0.147) did not significantly differ between the two groups (Table 3). Among all included patients, 15.1% (8/53) of those in the modeling-guided ablation group and 18.2% (10/55) of those in the empirical ablation group underwent cardioversion to control AAD resistant recurred atrial arrhythmias (Table 3). Repeat ablation procedures were performed in 14 patients (11.3% in the modeling-guided ablation group vs. 14.5% in the empirical ablation group, *p* = 0.622), and reconnected PV potentials were found in 21.4% (3/14) in the modeling-guided ablation group and 42.9% (6/14) in the empirical ablation group (*p* = 0.240).

## Discussion

### Main Findings

In this study, we generated a highly efficient patient-specific computational AF modeling, which could be applied to the clinical AF ablation procedure. In this first multi-center prospective randomized clinical trial of 118 patients with PeAF (77.8% with longstanding PeAF), the modeling-guided ablation group applied the lesion set of the fastest virtual AF termination after comparing five different virtual lesion sets. During the mean 31.5 months follow-up, the computational modeling-guided ablation results were superior to the empirical catheter ablation results regarding the rhythm outcome. The rhythm outcome of the computational modeling-guided ablation showed better trends in patients with a less structurally remodeled atrium.

### PeAF Ablation: Unanswered Continuous Challenge

Atrial fibrillation catheter ablation has been proven to reduce the AF burden, heart failure mortality (Marrouche et al., 2018), and stroke risk, and it also improves the cognitive and renal function (Takahashi et al., 2011; Calkins et al., 2012). Although the CPVI is a cornerstone lesion of AF ablation in both paroxysmal and PeAF (Verma et al., 2015), the 1-year rhythm outcome is not satisfactory in patients with PeAF with multiple extra-PV triggers (Pak et al., 2006). Thus, an empirical extra-PV ablation for a substrate modification has been applied in patients with PeAF for many years and is known to be beneficial (Wynn et al., 2014). However, the recent prospective randomized clinical trial, STAR-AF2, failed to prove its usefulness (Verma et al., 2015). Therefore, personalized extra-PV AF rotors or drivers have been traced by clinical investigators (Narayan et al., 2014). We also demonstrated the effectiveness of a virtual ablation targeting AF spiral wave reentries represented by a high dominant frequency area in the computational modeling study (Hwang et al., 2016), but the majority were non-stationary rotors that were affected by the local conduction velocity (Narayan et al., 2014). Currently, catheter ablation targeting these non-stationary hypothetical triggers is controversial in patients with PeAF (Calkins et al., 2012). Rather, we need a more reproducible and clinically feasible sophisticated mapping technique reflecting the patient-specific anatomy and electrophysiology that affects the AF wave-dynamics.

### Current Technology of Computational AF Modeling

Cardiac computational modeling has a growing role and allows for the non-invasive identification of spiral wave reentries in AF (Lim et al., 2017). Although the contemporary clinical 3D electroanatomical mapping system has enabled contact electrogram-based mapping of the atrium in detail, it is not possible to identify non-stationary AF drivers by point-to-point catheter mapping. The entire chamber mapping, such as focal impulse and rotor mapping (FIRM) (Narayan et al., 2014) or panoramic mapping (Haissaguerre et al., 2014), has a limitation in its spatial resolution. In recent years, high-performance computational simulation technology has enabled patient-specific AF modeling that reflects MRI-defined fibrosis, the fiber orientation, and the tissue thickness, and has been clinically applied (Supplementary Table 1) (Jacquemet, 2015). With this sophisticated computational modeling, high-density entire chamber mapping of AF, reflecting a personalized anatomy and histology, is possible. However, the long computing time is the major limitation for the application of this sophisticated and realistic AF modeling to clinical practice. Therefore, we generated a high-speed high-density (half million nodes) chamber-centric AF computational modeling integrated with the patient-specific atrial anatomy. A graphics processing unit system was used to sufficiently improve the computational speed to be applicable for clinical AF ablation (Hwang et al., 2014). We also validated its reproducibility in a retrospective clinical study (Hwang et al., 2014) and feasibility (Shim et al., 2017) and efficacy in this prospective clinical study. Although it is technically possible, we did not incorporated patient-specific atrial fibrosis or fiber orientation in this study because of computational speed issue. In order to apply more sophisticated patient-specific information that is acquired during the on-site procedure, the technological innovation that can increase the computation speed is expected. However, AF is a multifactorial systemic degenerative disease and its rhythm outcome can be affected by metabolic factors, such as obesity (Mahajan et al., 2018), pericardial fat volume (Kim et al., 2014) and obstructive sleep apnea (Dimitri et al., 2012). Since CUVIA computational modeling reflects the characteristics of patients’ atrium proper, its predictive value might be higher in non-obese patients with less remodeled atria.

### Limitations and Future Clinical Applications

Although we applied the patient-specific atrial anatomy for our AF modeling study, it was a monolayer homogeneous model. This modeling study is only related to anatomical factor and their clear relation with AF activation patterns remains unclear. While the atrial thickness variations, epicardial conduction (Hansen et al., 2016), and myofiber orientation could affect the cardiac wave propagation (Hansen et al., 2015), the wave propagation pattern of the monolayer model is very similar to that of the bilayer model (Labarthe et al., 2014). Reflection of the atrial wall thickness may improve the cardiac wave dynamics analyses as well as the RF energy titration during clinical AF ablation procedures in the future. A more realistic AF computational modeling application is under way, applying the contact electrogram voltage and local activation pattern acquired during the AF ablation procedure and the atrial wall thickness obtained from atrial imaging. Therefore, it is challenging to further reduce the computing simulation time to generate an upgraded system applicable in *in situ* procedures. Although we used irrigated-tip catheters for clinical AF ablation, we did not use contact-force technology nor ablation lesion index in this study. Additionally, although patients were followed as the guideline (Calkins et al., 2012), 24-h Holter recording at 3, 6, 12 months is relatively overestimates the success, therefore, careful interpretation is needed (Verma et al., 2013). The lack of a continuous rhythm recording, such as implantable loop recorder, may alter the outcome by detection of silent and/or short AF recurrences. Although randomization was performed by a central randomization service independent of investigators, the number of patients with AADs after the procedure tend to be more in computational modeling-guided ablation group than in empirical ablation group; however, the difference was not statistically significant (*p* > 0.10). Therefore, careful interpretation is needed in this respect. In order to make this study outcome more appealing and realistic, a larger study population will be needed. The individuals in this study were limited to PeAF patients, and the outcome of this study cannot be extrapolated to other types of AF or the entire AF population.

## Conclusion

We tested the feasibility and efficacy of highly efficient patient-specific computational AF modeling in a multi-center prospective randomized clinical trial. During the mean 31.5 months follow-up, the computational modeling-guided ablation was superior to the empirical catheter ablation regarding the rhythm outcome. The rhythm outcome of the computational modeling-guided ablation showed better trends in patients with a less structurally remodeled atrium.

## Data Availability Statement

The datasets generated for this study will not be made publicly available. This study was analyzed with the individual patient data from different participated centers.

## Ethics Statement

The studies involving human participants were reviewed and approved by the institutional review board of each participating center (described on manuscript). The patients/participants provided their written informed consent to participate in this study.

## Author Contributions

H-NP conceived and designed the study. I-SK, JS, HY, T-HK, BJ, S-HK, Y-SO, G-BN, YO, SO, Y-HK, and H-NP acquired the data. I-SK, JS, MH, J-SU, and BL analyzed the data. I-SK, ES, and H-NP interpreted the data. I-SK, JS, and MH drafted the work. All authors contributed in revising the work, approved the final version to be published, and agreed to be accountable for all aspects of the work.

## Conflict of Interest

The authors declare that the research was conducted in the absence of any commercial or financial relationships that could be construed as a potential conflict of interest.
