# Supplementary material for: Clinical Usefulness of Computational Modeling-Guided Persistent Atrial Fibrillation Ablation: Updated Outcome of Multicenter Randomized Study
Source: Front Physiol. 2019 Dec 17;10:1512. doi: 10.3389/fphys.2019.01512 (PMC6928133; doi:10.3389/fphys.2019.01512)
Supplement: Supplementary file 1 [file Table_1.DOCX]

**Supplementary Table.** Cutting edge virtual AF simulation

| Reference | Geometry | Heterogeneous  atrial characteristics | Computing time  to simulate 1 sec |
| --- | --- | --- | --- |
| Trayanova et al.(Trayanova et al., 2018) | MRI-derived  (CARP software since 2003 *)  Electrical mesh generation from segmented MRI images | Fiber orientation  (image-based estimation)  Fibrosis acquired from MRI | 47.33 min  (Intel X5660 CPU, 1,638,120 nodes) |
| Roney et al.(Roney et al., 2018) | MRI-derived  (using MUSIC software †)  SA node structure applied  Bilayer model | Fiber orientation  Fibrosis modeling | NA |
| Loewe et al.(Loewe et al., 2016) | MRI-derived  Homogeneous wall thickness (2.5-3mm)  Bilayer model | Fiber orientation  (Patient-specific) | NA |
| Jacquemet(Jacquemet, 2015) | Geometry from patient’s imaging  Cubic mesh formation  Uniform wall thickness | Fiber orientation  (Patient-specific) | NA |
| CUVIA 2.5(Lim et al., 2017) | CT-based geometry  Monolayer model | Fiber orientation  (image-based estimation)  Fibrosis acquired from the clinical voltage map | 1.2 min  (Intel i5 6600 + GPU Titan V, 500,000 nodes) |

* CARP: Cardiac arrhythmias research package

† MUSIC: Multi-modality Platform for Specific Imaging in Cardiology

**References**

Jacquemet, V. (2015). Modeling left and right atrial contributions to the ECG: A dipole-current source approach. *Computers in biology and medicine* 65, 192-199. doi:10.1016/j.compbiomed.2015.06.007.

Lim, B., Hwang, M., Song, J. S., Ryu, A. J., Joung, B., Shim, E. B., Ryu, H., and Pak, H. N. (2017). Effectiveness of atrial fibrillation rotor ablation is dependent on conduction velocity: An in-silico 3-dimensional modeling study. *PloS one* 12, e0190398. doi:10.1371/journal.pone.0190398.

Loewe, A., Krueger, M. W., Holmqvist, F., Dossel, O., Seemann, G., and Platonov, P. G. (2016). Influence of the earliest right atrial activation site and its proximity to interatrial connections on P-wave morphology. *Europace : European pacing, arrhythmias, and cardiac electrophysiology : journal of the working groups on cardiac pacing, arrhythmias, and cardiac cellular electrophysiology of the European Society of Cardiology* 18, iv35-iv43. doi:10.1093/europace/euw349.

Roney, C. H., Williams, S. E., Cochet, H., Mukherjee, R. K., O'Neill, L., Sim, I., Whitaker, J., Razeghi, O., Klein, G. J., Vigmond, E. J., O'Neill, M., and Niederer, S. A. (2018). Patient-specific simulations predict efficacy of ablation of interatrial connections for treatment of persistent atrial fibrillation. *Europace : European pacing, arrhythmias, and cardiac electrophysiology : journal of the working groups on cardiac pacing, arrhythmias, and cardiac cellular electrophysiology of the European Society of Cardiology* 20, iii55-iii68. doi:10.1093/europace/euy232.

Trayanova, N. A., Boyle, P. M., and Nikolov, P. P. (2018). Personalized Imaging and Modeling Strategies for Arrhythmia Prevention and Therapy. *Current opinion in biomedical engineering* 5, 21-28. doi:10.1016/j.cobme.2017.11.007.
